# A Stochastic Differential Equation Model for the Spread of HIV amongst People Who Inject Drugs

**DOI:** 10.1155/2016/6757928

**Published:** 2016-03-09

**Authors:** Yanfeng Liang, David Greenhalgh, Xuerong Mao

**Affiliations:** Department of Mathematics and Statistics, University of Strathclyde, Glasgow G1 1XH, UK

## Abstract

We introduce stochasticity into the deterministic differential equation model for the spread of HIV amongst people who inject drugs (PWIDs) studied by Greenhalgh and Hay (1997). This was based on the original model constructed by Kaplan (1989) which analyses the behaviour of HIV/AIDS amongst a population of PWIDs. We derive a stochastic differential equation (SDE) for the fraction of PWIDs who are infected with HIV at time. The stochasticity is introduced using the well-known standard technique of parameter perturbation. We first prove that the resulting SDE for the fraction of infected PWIDs has a unique solution in (0, 1) provided that some infected PWIDs are initially present and next construct the conditions required for extinction and persistence. Furthermore, we show that there exists a stationary distribution for the persistence case. Simulations using realistic parameter values are then constructed to illustrate and support our theoretical results. Our results provide new insight into the spread of HIV amongst PWIDs. The results show that the introduction of stochastic noise into a model for the spread of HIV amongst PWIDs can cause the disease to die out in scenarios where deterministic models predict disease persistence.

## 1. Introduction

HIV (human immunodeficiency virus) is a deadly and infectious* Lentivirus* which attacks and weakens the immune system by especially attacking the CD4 cells. As a result, HIV causes AIDS (acquired immune seficiency syndrome). Since the first discovery of HIV in 1981, it has already infected almost 78 million people with about 39 million lives having been taken [1]. Despite the massive improvement in technology and medical equipment, we are still unable to fully find a cure for the HIV virus. In 2014, according to the reports by the World Health Organization, there were still approximately 36.9 million people living with HIV, with around 2 million new cases globally [2]. In order to control the epidemic, it is crucial to understand the dynamical behaviour of HIV and how it spreads within our community. There are various routes by which HIV can be transmitted, for example, transmission via unprotected sexual intercourse, vertically from infected mothers to their unborn children and people who inject drugs (PWIDs) sharing contaminated needles. Amongst all the possible routes of HIV transmission, PWIDs have become a significant risk group with around 3 million of them living with HIV [3]. For every 10 new cases of HIV infection, on average, one of them is caused by injecting drug use. In regions of Central Asia and Eastern Europe, injecting drug use accounts for 80 percent of HIV infections [3]. As a result, in this paper, we will focus on looking at this particular risk group.

Over the past years, mathematical models have been used successfully to analyse and predict the dynamical behaviour in biological systems. The first mathematical model for the spread of HIV and AIDS amongst PWIDs in shooting galleries was created by Kaplan [4], where a shooting gallery is a place for PWIDs to purchase and inject drugs. Kaplan incorporated many factors into his model such as the injection equipment sharing rate and the effect of cleaning injection equipment in order to better understand how HIV is transmitted within this type of community. Based on the original model created in [4], Greenhalgh and Hay [5] modified the model by changing some of the assumptions made by Kaplan to make them more realistic. These assumptions include having different visiting rates to the shooting galleries for PWIDs who have been diagnosed positive for the HIV virus and thus may have been advised to stop sharing injections and for those who either are not HIV positive or are but do not know it. The modified Kaplan model in [5] also allows for the possibility that an infected PWID may not always leave a needle infected before cleaning, as well as introducing different transmission probabilities for flushed and unflushed needles. The term “flushing” refers to the process where an infectious piece of injecting equipment is used by an uninfected PWID and thus after injecting the syringe is left uninfected. The HIV model that we will be looking at in this paper is based on the modified Kaplan model given in [5]. There have been many papers that have already looked at the connection between the spread of HIV and PWIDs [6–11]. However the models used are all deterministic models.

The real world is not deterministic, and there are many factors that can influence the behaviour of a disease and thus it is not always possible to predict with certainty what would happen. Consequently, a stochastic model would be more appropriate. Furthermore, a stochastic model also possesses many useful unique properties such as being able to calculate the probability that an endemic will not occur and the expected duration of an endemic. By running a stochastic model many times, we can also build up a distribution of the possible outcomes which allows us to identify the number of infectives at a particular time *t*, whereas for a deterministic model we will only get a single outcome. HIV infection is a behavioural disease and thus there are many environmental factors that can influence the spread of HIV. Rhodes et al. [12] have mentioned in detail how factors such as injecting environments, social network, and neighbourhood deprivation and poverty can affect the spread of HIV amongst PWIDs. There are also other papers which emphasised how the dynamical behaviour of HIV is highly correlated with other factors [13–15]. Consequently, it is crucial for us to understand how HIV would spread under those environmental influences, especially amongst PWIDs. In this case, a stochastic model would be useful. There is also natural biological variation within people in their response to HIV. Using a stochastic model with environmental perturbation in the disease transmission parameter as we will do is one way to include this.

The stochastic aspects of the HIV model have been studied by many authors. For example, in [16], Dalal et al. considered a stochastic model for internal HIV dynamics. They incorporated environmental stochasticity into their model by using the standard technique of parameter perturbation. They proved that the solution (representing the concentrations of uninfected cells, infected cells, and virus particles) is nonnegative and have looked at the stability aspect of their model by establishing the conditions required in order for the numbers of infected cells and virus particles to tend asymptotically to zero exponentially almost surely. Ding et al. [13] looked at a stochastic model for AIDS transmission and control taking into consideration the treatment rate of HIV patients. They have also examined the effect that knowledge, attitude, and behaviour of patients have on the spread of AIDS. Tuckwell and Le Corfec [17] used a stochastic model to analyse the behaviour of HIV-1 but focus only on the early stage after infection. Dalal et al. [18] have also used a stochastic model to look at another aspect of HIV. Once again, by using parameter perturbation, they introduced environmental randomness into their HIV model which allows them to examine the effect that condom use has on the spread of AIDS among a homogeneous homosexual population which is split into distinct risk groups according to the tendency of individuals to use condoms. Peterson et al. [19] constructed a population-based simulation of a community of PWIDs using the Monte Carlo technique. Greenhalgh and Lewis [20] modelled the spread of disease using a set of behavioural assumptions due to Kaplan and O'Keefe [10]. They use a branching process approximation to show that if the basic reproduction number *R*_0_ is less than or equal to unity then the disease will always go extinct. They calculate an expression for the probability of extinction. They discuss an extended model which incorporates a three-stage incubation period and again examine a branching process approximation. They then compare them to investigate whether the deterministic model provides a good approximation to the simulated stochastic model. Although there have been many papers that looked at the stochastic aspect of the spread of HIV, as far as we know there are not many studies that focus on the stochastic aspect of the spread of HIV amongst PWIDs despite this particular risk group being responsible for many new HIV cases around the world. Thus it is crucial for us to examine the effect of environmental noise on this type of community.

Inspired by the model constructed in [5], in this paper we will introduce environmental stochasticity into the model by parameter perturbation which is a standard technique in stochastic population modelling [16, 18, 21, 22]. To the best of our knowledge, this is the first paper which examines the effect that environmental stochasticity has on the dynamical behaviour of the modified Kaplan model [5]. The techniques used in this paper are inspired by the work done in [21]. The paper is organised as follows. In the next section, we will describe the formulation of the stochastic HIV model amongst PWIDs. In Section 3, we shall prove the existence of a unique nonnegative solution. In Sections 4 and 5, we will investigate two of the main important properties of any biological system, namely, the conditions required for extinction and persistence, respectively. Then in Section 6, we shall show that there exists a stationary distribution for our system. Finally, we will perform some numerical simulations with realistic parameter values to verify the results.

## 2. The Stochastic HIV Model 

Throughout this paper, we let (*Ω*, *ℱ*, {*ℱ*_*t*_}_*t*≥0_, *ℙ*) be a complete probability space with filtration {*ℱ*_*t*_}_*t*≥0_ satisfying the usual conditions (i.e., it is increasing and right continuous while *ℱ*_0_ contains all *ℙ*-null sets). Let us consider the following deterministic HIV model, which has been constructed by Greenhalgh and Hay [5] based on the model of Kaplan [4]. Define the following parameters:

 
*λ*_1_: shooting gallery visiting rate for susceptible PWIDs and the PWIDs who are infected but do not know they are infected.
 
*λ*_2_: shooting gallery visiting rate for infected PWIDs who know that they are infected.
 
*P*_1_: probability that the needle is flushed and the PWID is infected.
 
*P*_2_: probability that the needle is flushed and the PWID remains uninfected.
 
*P*_3_: probability that the PWID becomes infected without the needle being flushed.
 
*P*_4_: probability that the PWID remains uninfected and the needle is not flushed.
 
*ϕ*_1_: probability that an infected PWID leaves uninfected a syringe that was initially uninfected.
 
*θ*_1_: probability that an infected PWID leaves uninfected a syringe that was initially infected.
 
*ξ*: fraction of all PWIDs (susceptible or not) who bleach their injection equipment after use.
 
*γ*: gallery ratio, where *γ* = *n*/*m* and *n* represents the PWID population and *m* represents the number of shooting galleries or syringes that each PWID visits at random.
 
*p*: probability that infected PWIDs know that they are infected.
 
*μ*: per capita rate at which infected PWIDs cease to share injection equipment (including those who cease sharing because of developing AIDS).

Note that *P*_1_ + *P*_2_ + *P*_3_ + *P*_4_ = 1.

Define the following new composite parameters:

(1) σ=λ11−p+λ2pγ1−ξ1−ϕ1,η=λ11−p+λ2pγξ+θ11−ξ,ρ=λ1γ1−1−ξ1−P1−P2,υ=λ1P1+P3.

In the expression for *σ* the factor (1 − *ξ*)(1 − *ϕ*_1_) represents the probability that an initially uninfected syringe is left infected and not cleaned by an infected PWID. The term *λ*_1_(1 − *p*) + *λ*_2_ *p* represents the average rate at which an infected PWID visits syringes. Hence $\sigma =\gamma \bar{\sigma }$, where *γ* = *n*/*m* is the gallery ratio and $\bar{\sigma }$ is the rate at which an infected PWID visits syringes multiplied by the probability that he or she leaves an uninfected syringe infected after use. Similarly $\eta =\gamma \bar{\eta }$, where $\bar{\eta }$ is the rate at which an infected PWID visits syringes multiplied by the probability that he or she leaves an infected syringe uninfected after use.

*λ*_1_ represents the rate at which a susceptible PWID visits syringes and 1 − (1 − *ξ*)(1 − *P*_1_ − *P*_2_) represents the probability that an initially infected syringe is left uninfected after use by that PWID. Hence $\rho =\gamma \bar{\rho }$, where $\bar{\rho }$ is the rate at which a susceptible PWID visits syringes multiplied by the probability that he or she leaves an infected syringe uninfected after use. *υ* represents the rate at which a susceptible PWID visits syringes multiplied by the probability that he or she becomes infected given that the syringe which they visit is infected. Thus *υβ* represents the rate at which a susceptible PWID visits syringes and becomes infected. *υ* can thus be regarded as the “potential” infection rate of a susceptible PWID.

Let *π*(*t*) and *β*(*t*) denote the proportion of infected PWIDs and proportion of infected needles, respectively. Thus the absolute numbers of infected PWIDs and infected needles are *nπ*(*t*) and *mβ*(*t*). The spread of the disease amongst syringes can be described by the following differential equation:

(2) dmβtdt=nπtσ¯1−βt−nπtη¯βt−n1−πtρ¯βt.

Dividing by *m*,

(3) dβtdtπtσ1−βt−πtηβt−1−πtρβt=πtσ−τβt−1−πtρβt,

where

(4) $$\begin{array}{c} \tau =\sigma +\eta =\gamma \left(\;\bar{\sigma }+\bar{\eta }\;\right), \end{array}$$

is the gallery ratio multiplied by the rate at which an infected PWID visits syringes multiplied by the sum of the probability that he or she leaves an uninfected syringe infected after use plus the probability that he or she leaves an infected syringe uninfected after use.

The spread of the disease amongst PWIDs can be described by the differential equation:

(5) $$\begin{array}{c} \frac{d\left(n\pi \left(t\right)\right)}{dt}=n\left(\;1-\pi \left(\;t\;\right)\;\right)\upsilon \beta \left(\;t\;\right)-\mu \left(\;n\pi \left(\;t\;\right)\;\right). \end{array}$$

Dividing by *n*,

(6) $$\begin{array}{c} \frac{d\pi \left(t\right)}{dt}=\left(\;1-\pi \left(\;t\;\right)\;\right)\upsilon \beta \left(\;t\;\right)-\mu \pi \left(\;t\;\right). \end{array}$$

So in summary the equations describing the deterministic HIV model are

(7) dβtdt=πtσ−τβt−1−πtρβt,dπtdt=1−πtυβt−μπt.

Greenhalgh and Hay define the basic reproduction number for the modified Kaplan model to be

(8) $$\begin{array}{c} R_{0}^{D}=\frac{\upsilon \sigma }{\rho \mu }, \end{array}$$

where in Section  4.5 of their paper [5] they have shown in detail that it corresponds to the usual biological definition, that is, the expected number of secondary infected PWIDs (infected PWIDs who became infected from sharing a syringe with the original infected PWID) caused during his or her entire infectious period by a single newly infected PWID entering a disease-free population at equilibrium. They also point out that it is also the expected number of secondary infected needles caused by a single newly infected needle entering the disease-free population at equilibrium.

Greenhalgh and Hay then show that the disease dies out if *R*_0_^*D*^ < 1 or *R*_0_^*D*^ = 1 and *τ* > *ρ*. If *R*_0_^*D*^ > 1 there are two possible equilibria, one with no disease present and the other with disease present. Thus this value of *R*_0_^*D*^ clearly satisfies the usual properties of the deterministic threshold value in epidemic models. There is a unique endemic equilibrium

(9) β∗=στ1−ρμσυ,π∗=συ−ρμμτ+συ−ρμ.

If *R*_0_^*D*^ > 1 then the unique endemic equilibrium is locally stable. If *τ* > *ρ* and *R*_0_^*D*^ > 1, then *β*(*t*) → *β* ^*∗*^ and *π*(*t*) → *π* ^*∗*^ as *t* → *∞* provided that *π*(0) > 0 or *β*(0) > 0.

Note that the average rate at which PWIDs leave the sharing, injecting population is around 0.25/year [5] so, on average, they each share for four years. *P*_1_ + *P*_3_, the probability of HIV transmission to a susceptible PWID on making a single injection with an infected syringe, is quite small (e.g., one estimate is 0.01 [5]). On the other hand, PWIDs are injected every few days which is a much shorter timescale than the demographic epidemiological changes which take several years. Hence changes in the fraction of syringes infected will typically happen a lot faster than changes in the fraction of PWIDs infected.

Thus over an intermediate timescale it may be reasonable to assume that *π*(*t*) is approximately constant in the last equation of (7). Hence *β*(*t*) will approach its equilibrium value from this equation

(10) $$\begin{array}{c} \frac{\pi \left(t\right)\sigma }{\pi \left(t\right)+\rho -\rho \pi \left(t\right)}. \end{array}$$

By substituting (10) into the second equation in (7) we deduce that

(11) $$\begin{array}{c} \frac{d\pi \left(t\right)}{dt}=\frac{\left(1-\pi \left(t\right)\right)\pi \left(t\right)\upsilon \sigma }{\pi \left(t\right)\tau +\rho -\pi \left(t\right)\rho }-\mu \pi \left(\;t\;\right). \end{array}$$

A similar technique of reducing the dimensions of the model by assuming that the needle equations are at equilibrium is used in models of variable infectivity of spread of HIV amongst PWIDs discussed by Greenhalgh and Lewis [8, 11] and Corson et al. [23, 24].

In this paper, we introduce environmental stochasticity into system (11) by replacing the parameter *υ* by $\upsilon +\bar{\upsilon }(dB\left(t\right)/dt)$, where *B*(*t*) is a Brownian motion and $\bar{\upsilon }>0$ is the intensity of the noise which is associated with the potential rate of infection *υ*. It is therefore clear that the total number of new PWIDs infected during the small time interval [*t*, *t* + *dt*) is normally distributed with mean,

(12) $$\begin{array}{c} \frac{n\left(1-\pi \left(t\right)\right)\pi \left(t\right)\upsilon \sigma }{\pi \left(t\right)\tau +\rho -\pi \left(t\right)\rho }dt, \end{array}$$

and variance,

(13) $$\begin{array}{c} \frac{n^{2}{\left(1-\pi \left(t\right)\right)}^{2}\pi {\left(t\right)}^{2}{\bar{\upsilon }}^{2}\sigma^{2}}{{\left(\pi \left(t\right)\tau +\rho -\pi \left(t\right)\rho \right)}^{2}}dt. \end{array}$$

Notice that both of this mean and variance tend to zero as *dt* goes to zero which is a biologically desirable property. This is a standard technique of introducing random noise in stochastic modelling [16, 18, 25–28] and corresponds to some stochastic environmental factor acting on each individual in the population.

To justify why simple white noise is appropriate for our model suppose that we consider a timescale on which *β*(*t*) and *π*(*t*) are approximately constant. We consider the changes in a small time interval [*t*, *t* + *T*_0_) and divide it into a series of *n*_0_ equal width subintervals [*t*, *t* + *T*, [*t* + *T*, *t* + 2*T*,…, [*t* + (*n*_0_ − 1)*T*, *t* + *n*_0_ *T*), where *n*_0_ *T* = *T*_0_ and *n*_0_ is very large. Then the numbers of new infections caused by a single susceptible PWID visiting one infected syringe during each of the subintervals [*t*, *t* + *T*, [*t* + *T*, *t* + 2*T*,…, [*t* + (*n*_0_ − 1)*T*, *t* + *n*_0_ *T*) are identically distributed random variables, say, with common mean *μ*_0_ and common variance *σ*_0_^2^. We assume that *σ*_0_^2^ < *∞*. So by the Central Limit Theorem the total number of PWIDs who visit infected syringes and become infected in [*t*, *t* + *n*_0_ *T*) = [*t*, *t* + *T*_0_) is approximately normally distributed with mean *n*_0_ *μ*_0_ and variance *n*_0_ *σ*_0_^2^. Moreover keeping *T* fixed and doubling *T*_0_ doubles *n*_0_ thus the mean and variance of the number of susceptible PWIDs who become infected from visiting an infected syringe in [*t*, *t* + *T*_0_) are both proportional to *T*_0_. Hence it is appropriate to consider simple white noise where the mean number of infections in [*t*, *t* + *dt*) caused by a given susceptible PWID visiting a given infected syringe is *υdt* (hence proportional to *dt*) the same as in the deterministic model and the variance of this number is also proportional to *dt*.

As a result, we obtain the following SDE HIV model:

(14) dπt=1−πtπtυσπtτ+ρ−πtρ−μπtdt+1−πtπtυ¯σπtτ+ρ−πtρdBt.

The reason why we chose to perturb the parameter *υ* corresponding to the total rate at which PWIDs visit syringes and potentially become infected is because as it multiplies the term *π*(1 − *π*) in (11) it is a key parameter in the transmission of HIV amongst PWIDs and we thought that this would be the most interesting and important parameter when analysing the effect that environmental noise would have on the spread of HIV.

There are some environmental factors which can cause a perturbation in *υ*, for example, natural biological variation between people and between HIV viruses. These factors affect the probability *P*_1_ + *P*_3_ of HIV transmission to a susceptible PWID. It is possible that environmental noise causes variation in other parameters too, but it would be quite complicated to include these as well. Analysis of the model with environmental stochasticity in *υ* provides theoretical insight into the behaviour of the model. A similar approach of introducing environmental stochasticity into only the disease transmission parameter was discussed in stochastic studies of epidemic models by Ding et al. [13], Gray et al. [21], Lu [25], Tornatore et al. [29], and others.

For the rest of the paper, we shall focus on analysing the SDE HIV model (14). Throughout this paper, unless stated otherwise, we shall assume that the unit of time is one day.

## 3. Existence of Unique Nonnegative Solution

Before we begin to investigate the dynamical behaviour of the SDE HIV model (14), it is important for us to show whether this SDE has a unique global nonnegative solution. It is well-known that, in order for an SDE to have a unique global solution for any given initial value, the coefficients of the equation are generally required to satisfy the linear growth condition and the local Lipschitz conditions [30]. It is clear that our coefficients in (14) satisfy the linear growth condition and they are locally Lipschitz continuous. As a result there is a unique, nonexplosive solution to (14). The following theorem shows that the solution remains in (0,1) if it starts there.

**Theorem 1 .**

For any given initial value *π*(0) = *π*_0_ ∈ (0,1), the SDE HIV model (14) has a unique global nonnegative solution *π*(*t*)∈(0,1) for all *t* ≥ 0 with probability one; namely,

(15) $$\begin{array}{c} P\left\{\;\pi \left(\;t\;\right)\in \left(\;0,1\;\right),\;\;\forall t\geq 0\;\right\}=1. \end{array}$$

**Proof**

For any given initial value *π*_0_ ∈ (0,1), there is a unique global solution *π*(*t*) for *t* ≥ 0. Let *k*_0_ ≥ 0 be sufficiently large so that *π*_0_ lies within the interval (1/*k*_0_, 1 − (1/*k*_0_)). Then for each integer *k* ≥ *k*_0_, define the stopping time

(16) $$\begin{array}{c} \tau_{k}=\inf\left\{\;t\geq 0:\pi \left(\;t\;\right)\notin \left(\;\frac{1}{k},1-\left(\;\frac{1}{k}\;\right)\;\right)\;\right\}, \end{array}$$

where inf⁡*∅* = *∞*. It is easy to see that *τ*_*k*_ is increasing as *k* → *∞*. Let us also define *τ*_*∞*_ = lim_*k*→*∞*_ *τ*_*k*_. To complete the proof, we need to show that *τ*_*∞*_ = *∞* a.s. We will carry this proof out by contradiction. Let us therefore assume that the statement is false and thus there exists a pair of constants *T* > 0 and *ε* ∈ (0,1) such that

(17) $$\begin{array}{c} P\left\{\;\tau_{\infty }\leq T\;\right\}>\varepsilon . \end{array}$$

Hence, there is an integer *k*_1_ ≥ *k*_0_ such that

(18) $$\begin{array}{c} P\left\{\;\tau_{k}\leq T\;\right\}>\varepsilon \;\forall k\geq k_{1}. \end{array}$$

Let us define a function *V* : (0,1) → *ℝ*,

(19) $$\begin{array}{c} V\left(\;x\;\right)=\frac{1}{x}+\frac{1}{1-x}. \end{array}$$

Now by Itô's formula, we have that, for any *t* ∈ [0, *T*] and *k* ≥ *k*_1_,

(20) $$\begin{array}{c} EV\left(\;\pi \left(\;t\wedge \tau_{k}\;\right)\;\right)=V\left(\;\pi_{0}\;\right)+E\overset{t\wedge \tau_{k}}{\underset{0}{\int }}LV\left(\;\pi \left(\;s\;\right)\;\right)ds, \end{array}$$

where *LV* : (0,1) → *ℝ* is defined by

(21) LVx=−1−xυσxxτ+ρ−xρ+xυσxτ+ρ−xρ1−x+μx−μx1−x2+1−x2υ¯2σ2xτ+ρ−xρ2x+x2υ¯2σ21−xxτ+ρ−xρ2.

Furthermore, it is easy to see that

(22) LVxυσmin⁡τ,ρ1−x+μx+υ¯2σ2min⁡τ,ρ21x+11−x≤CVx,

where

(23) $$\begin{array}{c} C=\left\{\;\left[\;\frac{\upsilon \sigma }{\min\left(\tau ,\rho \right)}\;\right]\vee \mu \;\right\}+\frac{{\bar{\upsilon }}^{2}\sigma^{2}}{\min{\left(\tau ,\rho \right)}^{2}}. \end{array}$$

Here *a*∨*b* denotes the maximum of *a* and *b*. By substituting this into (20), we have that for any *t* ∈ [0, *T*]

(24) $$\begin{array}{c} EV\left(\;\pi \left(\;t\wedge \tau_{k}\;\right)\;\right)\leq V\left(\;\pi_{0}\;\right)+C\overset{t}{\underset{0}{\int }}EV\left(\;\pi \left(\;s\wedge \tau_{k}\;\right)\;\right)ds. \end{array}$$

Then by using the Gronwall inequality we have that

(25) $$\begin{array}{c} EV\left(\;\pi \left(\;t\wedge \tau_{k}\;\right)\;\right)\leq V\left(\;\pi_{0}\;\right)e^{Ct}\leq V\left(\;\pi_{0}\;\right)e^{CT}. \end{array}$$

Let us set *Ω*_*k*_ = {*τ*_*k*_ ≤ *T*} for *k* ≥ *k*_1_, and so, by (18), we have that *ℙ*(*Ω*_*k*_) ≥ *ε*. For every *ω* ∈ *Ω*_*k*_, *π*(*τ*_*k*_, *ω*) equals either 1/*k* or 1 − (1/*k*) and thus *V*(*π*(*τ*_*k*_, *ω*)) ≥ *k*. Consequently we have that

(26) Vπ0εCTE1ΩkωVπτk,ω≥kPΩk≥εk.

Letting *k* → *∞*, we have a contradiction, where *∞* > *V*(*π*_0_)*ε* ^*CT*^ = *∞*. Therefore, our assumption at the beginning must be false and thus we obtained our desired result that *τ*_*∞*_ = *∞* a.s.

In this section we have managed to show that there exists a unique nonnegative global solution for the SDE HIV model (14) which remains in (0,1).

## 4. Extinction

When studying the dynamical behaviour of a population system, it is important for us to consider the conditions required in order for the HIV amongst PWIDs to die out, in other words when the disease will become extinct. We will split this proof into two parts, each considering two different scenarios of the noise intensity, namely, $\bar{\upsilon }.$ Before we begin the proof, let us recall the basic reproduction number for the deterministic model of Greenhalgh and Hay [5]:

(27) $$\begin{array}{c} R_{0}^{D}=\frac{\upsilon \sigma }{\rho \mu }, \end{array}$$

where all the parameters are defined as before.

For the stochastic model we define the stochastic basic reproduction number

(28) $$\begin{array}{c} R_{0}^{S}=R_{0}^{D}-\frac{{\bar{\upsilon }}^{2}\sigma^{2}}{2\rho^{2}\mu }. \end{array}$$

This is the deterministic basic reproduction number *R*_0_^*D*^ corrected for the effect of stochastic noise and plays a role in the stochastic model with many similarities to *R*_0_^*D*^ in the deterministic one.

**Theorem 2 .**

If the stochastic reproduction number

(29) $$\begin{array}{c} R_{0}^{S}=R_{0}^{D}-\frac{{\bar{\upsilon }}^{2}\sigma^{2}}{2\rho^{2}\mu }<1,\;{\bar{\upsilon }}^{2}\leq \frac{\upsilon \rho }{\sigma }, \end{array}$$

then, for any given initial value *π*(0) = *π*_0_ ∈ (0,1), the solution of (14) obeys

(30) limt→∞sup⁡1tlog⁡πtυσρ−υ¯2σ22ρ2−μ=μR0S−1<0a.s.

In other words, *π*(*t*) will tend to zero exponentially a.s. Thus the fraction of population that is infected with HIV at time *t* will approach zero.

**Proof**

Let us define a function *V*(*x*) = log⁡(*x*), where by Itô's formula we have that

(31) log⁡πt=log⁡π0+∫0tfπsds+∫0t1−πsυ¯σπsτ+ρ−πsρdBs.

Here *f* : (0,1) → *ℝ* is defined as

(32) fx=1−xυσxτ+ρ−xρ−μ−1−x2υ¯2σ22xτ+ρ−xρ2

(33) =υσφ+ρ−μ−υ¯2σ22φ+ρ2,

where *φ* = *xτ*/(1 − *x*). Moreover

(34) f′φ−υσφ+ρ2+υ¯2σ2φ+ρ3<−υσφ+ρ2+υρσφ+ρ3<0.

Hence *f*(*φ*) is a monotone decreasing function of *φ* for *φ* > 0, and thus we must have that

(35) $$\begin{array}{c} f\left(\;x\;\right)\leq {\left.\;f\left(\;x\;\right)\;\right|}_{\varphi =0}=\mu \left(\;R_{0}^{S}-1\;\right)<0, \end{array}$$

where *R*_0_^*S*^ is the stochastic reproduction number defined in Theorem 2. As a result, (31) becomes

(36) log⁡πt≤log⁡π0+tμR0S−1+∫0t1−πsυ¯σπsτ+ρ−πsρdBs.

This implies that

(37) limt→∞sup⁡1tlog⁡πt≤μR0S−1+limt→∞sup1t∫0t1−πsυ¯σπsτ+ρ−πsρdBs.

However, since

(38) $$\begin{array}{c} 0\leq \frac{\left(1-\pi \left(s\right)\right)}{\pi \left(s\right)\tau +\rho -\pi \left(s\right)\rho }\leq \frac{1}{\min\left(\tau ,\rho \right)}, \end{array}$$

then, by the large number theorem of martingales (e.g., [28]), we have that

(39) $$\begin{array}{c} \underset{t\to \infty }{lim}sup\frac{1}{t}\overset{t}{\underset{0}{\int }}\frac{\left(1-\pi \left(s\right)\right)\bar{\upsilon }\sigma }{\pi \left(s\right)\tau +\rho -\pi \left(s\right)\rho }dB\left(\;s\;\right)=0\;a.s. \end{array}$$

Hence, we have arrived at our desired result, where

(40) $$\begin{array}{c} \underset{t\to \infty }{lim}sup\frac{1}{t}\log\left(\;\pi \left(\;t\;\right)\;\right)\leq \mu \left(\;R_{0}^{S}-1\;\right)<0\;a.s. \end{array}$$

In other words, *π*(*t*) tends to zero exponentially a.s.

In Theorem 2, we have focused on discussing the extinction conditions for our SDE HIV model (14); we have considered a partial case, where the noise intensity satisfies the condition ${\bar{\upsilon }}^{2}\leq \upsilon \rho /\sigma$. In order to get a better picture of the dynamical behaviour of our SDE HIV model (14), it is important for us to investigate what happens to the population system when ${\bar{\upsilon }}^{2}>\upsilon \rho /\sigma$.

**Theorem 3 .**

If

(41) $$\begin{array}{c} R_{0}^{S}=R_{0}^{D}-\frac{{\bar{\upsilon }}^{2}\sigma^{2}}{2\rho^{2}\mu }<1,\;{\bar{\upsilon }}^{2}>\frac{\upsilon \rho }{\sigma }\vee \frac{\upsilon^{2}}{2\mu }, \end{array}$$

then, for any given initial value *π*(0) = *π*_0_ ∈ (0,1), the solution of (14) obeys

(42) $$\begin{array}{c} \underset{t\to \infty }{lim}\sup\frac{1}{t}\log\pi \left(\;t\;\right)\leq \frac{\upsilon^{2}}{2{\bar{\upsilon }}^{2}}-\mu <0\;a.s. \end{array}$$

In other words, *π*(*t*) will tend to zero exponentially a.s. Thus the fraction of the population that are infected with HIV at time *t* will become zero.

**Proof**

In order to simplify the computation, throughout this proof, we will be working with (33). It is easy to see that this function has a maximum turning point at

(43) $$\begin{array}{c} \varphi =\hat{\varphi }=\frac{{\bar{\upsilon }}^{2}\sigma }{\upsilon }-\rho . \end{array}$$

Note that, by substituting (43) back into the expression *φ* = *xτ*/(1 − *x*), we could easily obtain the same result as we would if we decided to work with the alternative function

(44) $$\begin{array}{c} x=\hat{x}=\frac{{\bar{\upsilon }}^{2}\sigma -\rho \upsilon }{\upsilon \left(\tau -\rho \right)+{\bar{\upsilon }}^{2}\sigma }, \end{array}$$

where $\hat{x}\in (0,1)$. Note also that $\hat{\varphi }>0$ by (41). Furthermore, by substituting the maximum turning point $\hat{\varphi }$ given in (43) into (33), we have that $f(x){|}_{\varphi =\hat{\varphi }}=\upsilon^{2}/2{\bar{\upsilon }}^{2}-\mu$ which is negative by condition (41). Therefore, arguing as before in Theorem 2, we have that

(45) log⁡πt≤log⁡π0+tυ22υ¯2−μ+∫0t1−πsυ¯σπsτ+ρ−πsρdBs,

which similarly implies that

(46) $$\begin{array}{c} \underset{t\to \infty }{lim}\sup\frac{1}{t}\log\left(\;\pi \left(\;t\;\right)\;\right)\leq \frac{\upsilon^{2}}{2{\bar{\upsilon }}^{2}}-\mu <0\;a.s. \end{array}$$

In other words, *π*(*t*) will also tend to zero exponentially a.s. for ${\bar{\upsilon }}^{2}>\upsilon \rho /\sigma \vee \upsilon^{2}/2\mu$ and thus we have completed the proof.

Note that *R*_0_^*S*^ < *R*_0_^*D*^, which implies that the condition for extinction is weaker in the stochastic case compared to the deterministic case. In addition, as $\bar{\upsilon }$ increases, the stochastic reproduction number *R*_0_^*S*^ will become smaller and thus it will be more likely for the HIV virus to die out for large noise intensity. As a result, this highlights the fact that environmental factors play an important role in the dynamical behaviour of HIV amongst PWIDs.

There is a gap in our results for *R*_0_^*S*^ < 1. We have not shown what will happen if *R*_0_^*S*^ < 1 and

(47) $$\begin{array}{c} \frac{\upsilon \rho }{\sigma }<{\bar{\upsilon }}^{2}<\frac{\upsilon^{2}}{2\mu }, \end{array}$$

but we conjecture that in this case the disease will die out a.s. This was confirmed by simulation.

## 5. Persistence

Another very important aspect of the behaviour of a dynamical system is the conditions for persistence. In this section we will discuss the persistence conditions required for our SDE HIV model (14).

**Theorem 4 .**

If

(48) $$\begin{array}{c} R_{0}^{S}=R_{0}^{D}-\frac{{\bar{\upsilon }}^{2}\sigma^{2}}{2\rho^{2}\mu }>1, \end{array}$$

then, for any given initial value *π*(0) = *π*_0_ ∈ (0,1), the solution of (14) satisfies

(49) limt→∞sup⁡πt≥ηa.s,

(50) limt→∞inf⁡πt≤ηa.s,

where

(51) $$\begin{array}{c} \eta =\frac{\upsilon \sigma -2\mu \rho +\sqrt{\upsilon^{2}\sigma^{2}-2\mu {\bar{\upsilon }}^{2}\sigma^{2}}}{2\mu \tau +\upsilon \sigma -2\mu \rho +\sqrt{\upsilon^{2}\sigma^{2}-2\mu {\bar{\upsilon }}^{2}\sigma^{2}}}>0, \end{array}$$

which is the unique root in (0,1) of the function

(52) $$\begin{array}{c} f\left(\;x\;\right)=\frac{\left(1-x\right)\upsilon \sigma }{x\tau +\rho -x\rho }-\mu -\frac{{\left(1-x\right)}^{2}{\bar{\upsilon }}^{2}\sigma^{2}}{2{\left(x\tau +\rho -x\rho \right)}^{2}}=0, \end{array}$$

defined in (32). In other words, the solution *π*(*t*) will persist and oscillate around the level *η* infinitely often with probability one.

**Proof**

Let us recall the function *f* : (0,1) → *ℝ* defined in (33). Throughout this proof, we will be working with this function in order to simplify the computation.

By setting *f*(*x*) = 0, we obtain one positive and one negative root, where the positive root is

(53) φ∗=12μυσ−2μρ2+4μυσρ−μρ2−0.5υ¯2σ2+υσ−2μρ>φ^,

where $\hat{\varphi }$ is the maximum turning point of (33) defined in (43). For the purpose of consistency, we will now substitute (53) into the expression *φ* ^*∗*^ = *x* ^*∗*^ *τ*/(1 − *x* ^*∗*^) to get that

(54) $$\begin{array}{c} x^{*}=\eta =\frac{\upsilon \sigma -2\mu \rho +\sqrt{\upsilon^{2}\sigma^{2}-2\mu {\bar{\upsilon }}^{2}\sigma^{2}}}{2\mu \tau +\upsilon \sigma -2\mu \rho +\sqrt{\upsilon^{2}\sigma^{2}-2\mu {\bar{\upsilon }}^{2}\sigma^{2}}}>\hat{x}, \end{array}$$

where *x* ^*∗*^ ∈ (0,1), and that $\hat{x}$ is the equivalent maximum turning point of (32) defined in (44). Moreover, it is easy to see that

(55) f0=υσρ−μ−υ¯2σ22ρ2>0,f1=−μ<0.

As a result we have that

(56) fx>0is  strictly  increasing  on  x∈0,0∨x^,

(57) fx>0is  strictly  decreasing  on  x∈0∨x^,x∗,

(58) fx<0is  strictly  decreasing  on  x∈x∗,1,

where $\hat{x}$ and *x* ^*∗*^ are defined as before. Let us now prove result (49) is true by contradiction. Assume that (49) is false and thus there must exist *ε* ∈ (0,1) small enough such that

(59) $$\begin{array}{c} P\left(\;\Omega_{1}\;\right)>\varepsilon , \end{array}$$

where *Ω*_1_ = {*ω* ∈ *Ω* : lim_*t*→*∞*_sup⁡*π*(*t*) ≤ *η* − 2*ε*}. Hence, for every *ω* ∈ *Ω*_1_, there is *T* = *T*(*ω*) > 0 such that

(60) $$\begin{array}{c} \pi \left(\;t,\omega \;\right)\leq \eta -\varepsilon \;for\;\;t\geq T\left(\omega \right). \end{array}$$

Clearly we can choose *ε* so small such that *f*(0) > *f*(*η* − *ε*). Therefore, from (56), (57), and (60), we have that *f*(*π*(*t*, *ω*)) > *f*(*η* − *ε*) for *t* ≥ *T*(*ω*). Let us now recall that, for *t* ≥ 0,

(61) log⁡πt=log⁡π0+∫0tfπsds+∫0t1−πsυ¯σπsτ+ρ−πsρdBs,

and then arguing as before, by the large number theorem of martingales, there is *Ω*_2_ ⊂ *Ω* with *ℙ*(*Ω*_2_) = 1, such that, for every *ω* ∈ *Ω*_2_,

(62) $$\begin{array}{c} \underset{t\to \infty }{lim}\frac{1}{t}\overset{t}{\underset{0}{\int }}\frac{\left(1-\pi \left(s\right)\right)\bar{\upsilon }\sigma }{\pi \left(s\right)\tau +\rho -\pi \left(s\right)\rho }dB\left(\;s\;\right)=0. \end{array}$$

Therefore by fixing any *ω* ∈ *Ω*_1_∩*Ω*_2_, then, for *t* ≥ *T*(*ω*),

(63) log⁡πt,ω≥log⁡π0+∫0Tωfπs,ωds+fη−εt−Tω+∫0t1−πsυ¯σπsτ+ρ−πsρdBs,ω,

which implies that

(64) $$\begin{array}{c} \underset{t\to \infty }{lim}\inf\frac{1}{t}\log\left(\;\pi \left(\;t,\omega \;\right)\;\right)\geq f\left(\;\eta -\varepsilon \;\right)>0, \end{array}$$

and thus we have that lim_*t*→*∞*_ *π*(*t*, *ω*) = *∞*. This is clearly a contradiction to (60). Thus, our assumption at the beginning must be wrong and therefore we obtained our desired result that

(65) $$\begin{array}{c} \underset{t\to \infty }{lim}\sup\pi \left(\;t\;\right)\geq \eta \;a.s. \end{array}$$

Similarly, we will prove (50) by assuming again that it is false and thus there must exist *δ* ∈ (0,1) such that

(66) $$\begin{array}{c} P\left(\;\Omega_{3}\;\right)>\delta , \end{array}$$

where *Ω*_3_ = {*ω* ∈ *Ω* : lim_*t*→*∞*_inf⁡*π*(*t*) ≥ *η* + 2*δ*}. Hence, for every *ω* ∈ *Ω*_3_, there is *τ* = *τ*(*ω*) > 0 such that

(67) $$\begin{array}{c} \pi \left(\;t,\omega \;\right)\geq \eta +\delta \;for\;\;t\geq \tau \left(\omega \right). \end{array}$$

Thus, we have that *f*(*π*(*t*, *ω*)) ≤ *f*(*η* + *δ*) for *t* ≥ *τ*(*ω*). Let us now fix any *ω* ∈ *Ω*_2_∩*Ω*_3_; then similarly to before, we would get that, for *t* ≥ *τ*(*ω*),

(68) log⁡πt,ω≤log⁡π0+∫0τωfπs,ωds+fη+δt−τω+∫0t1−πsυ¯σπsτ+ρ−πsρdBs,ω⟹limt→∞sup⁡1tlog⁡πt,ω≤fη+δ<0,

and thus

(69) $$\begin{array}{c} ⟹\underset{t\to \infty }{\lim}\pi \left(\;t,\omega \;\right)=0. \end{array}$$

This is clearly a contradiction to (67) and thus we have completed our proof.

In order to allow us to better understand the effect of the noise intensity $\bar{\upsilon }$ on the dynamical behaviour of our SDE HIV model (14) and its connection to the corresponding deterministic model (11), we have the following proposition.

**Proposition 5 .**

Suppose that *R*_0_^*S*^ > 1. Consider *η* as defined by (51) as a function of $\bar{\upsilon }$ for

(70) $$\begin{array}{c} 0<\bar{\upsilon }<\frac{\sqrt{2\rho \left(\upsilon \sigma -\mu \rho \right)}}{\sigma }=\hat{\upsilon }, \end{array}$$

and then *η* is strictly decreasing and

(71) $$\begin{array}{c} \underset{\bar{\upsilon }\to 0}{\lim}\eta =\frac{\upsilon \sigma -\mu \rho }{\upsilon \sigma -\mu \rho +\mu \tau }, \end{array}$$

which is the equilibrium state of the deterministic HIV model (11) and

(72) $$\begin{array}{c} \underset{\bar{\upsilon }\to \hat{\upsilon }}{\lim}\eta =\left\{\begin{array}{cc} 0, & if\;\;1\leq R_{0}^{D}\leq 2,\\ \frac{\upsilon \sigma -2\mu \rho }{\upsilon \sigma -2\mu \rho +\mu \tau }, & if\;\;R_{0}^{D}>2. \end{array}\right. \end{array}$$

In other words, the noise intensity $\bar{\upsilon }$ lies between the deterministic equilibrium value for *π*(*t*), namely, (*υσ* − *μρ*)/(*υσ* − *μρ* + *μτ*), and max(0, (*υσ* − 2*μρ*)/(*υσ* − 2*μρ* + *μτ*)). Furthermore, if the noise intensity decreases to zero, then *η* will increase to the deterministic equilibrium value. If *R*_0_^*D*^ is large, then *η* will be close to but beneath the deterministic equilibrium value for *π*(*t*).

**Proof**

Let us recall that

(73) $$\begin{array}{c} \eta =\frac{\upsilon \sigma -2\mu \rho +\sqrt{\upsilon^{2}\sigma^{2}-2\mu {\bar{\upsilon }}^{2}\sigma^{2}}}{2\mu \tau +\upsilon \sigma -2\mu \rho +\sqrt{\upsilon^{2}\sigma^{2}-2\mu {\bar{\upsilon }}^{2}\sigma^{2}}}. \end{array}$$

Then,

(74) dηdυ¯=−4μ2υ¯σ2τυ2σ2−2μυ¯2σ21/22μτ+υσ−2μρ+υ2σ2−2μυ¯2σ21/22.

Clearly, $d\eta /d\bar{\upsilon }<0$ since *σ* > 0 and thus *η* is strictly deceasing as $\bar{\upsilon }$ increases. By letting $\bar{\upsilon }$ tend to zero in the function for *η* defined above, we have the desired result given in (71). Moreover, as $\bar{\upsilon }\to \hat{\upsilon }$, we have that

(75) $$\begin{array}{c} \underset{\bar{\upsilon }\to \hat{\upsilon }}{\lim}\eta =\frac{\upsilon \sigma -2\mu \rho +\sqrt{\upsilon^{2}\sigma^{2}-2\mu {\hat{\upsilon }}^{2}\sigma^{2}}}{2\mu \tau +\upsilon \sigma -2\mu \rho +\sqrt{\upsilon^{2}\sigma^{2}-2\mu {\hat{\upsilon }}^{2}\sigma^{2}}}. \end{array}$$

The numerator of the above expression is equal to

(76) $$\begin{array}{c} \left|\;\upsilon \sigma -2\mu \rho \;\right|+\upsilon \sigma -2\mu \rho . \end{array}$$

As a result, if 1 ≤ *R*_0_^*D*^ ≤ 2, then it is obvious that ${lim}_{\bar{\upsilon }\to \hat{\upsilon }}\;\eta =0$. On the other hand, if *R*_0_^*D*^ > 2, then ${lim}_{\bar{\upsilon }\to \hat{\upsilon }}\;\eta =\left(\upsilon \sigma -2\mu \rho \right)/\left(\upsilon \sigma -2\mu \rho +\mu \tau \right)$. We have completed the proof.

## 6. Stationary Distribution

In this section, we will use the well-known Khasminskii theorem [31] to prove that there exists a stationary distribution for our stochastic HIV model (14). Before we begin, let us recall the conditions for the existence of a stationary distribution mentioned in [31].

**Lemma 6 .**

The SDE HIV model (14) has a unique stationary distribution if there is a strictly proper subinterval (*a*, *b*) of (0,1) such that *𝔼*(*τ*) < *∞* for all *π*_0_ ∈ (0, *a*)∪(*b*, 1), where

(77) τ=inf⁡t≥0:πt∈a,b,supπ0∈a¯,b¯⁡Eτ<∞for  every  interval  a¯,b¯⊂0,1.

Note that, in the original Khasminskii theorem, there is an additional condition which states that the square of the diffusion coefficient of the SDE HIV model (14), namely,

(78) $$\begin{array}{c} {\left(\;\frac{\left(1-\pi \left(t\right)\right)\pi \left(t\right)\bar{\upsilon }\sigma }{\pi \left(t\right)\tau +\rho -\pi \left(t\right)\rho }\;\right)}^{2}, \end{array}$$

is bounded away from zero for *π*(*t*)∈(*a*, *b*). However, recalling from the proof of Theorem 1, we have already shown that the denominator, (*π*(*t*)*τ* + *ρ* − *π*(*t*)*ρ*), is bounded away from zero (it is at least min⁡(*τ*, *ρ*)). Thus it is therefore clear that this condition holds for our model.

**Theorem 7 .**

If *R*_0_^*S*^ > 1, then the SDE HIV model (14) has a unique stationary distribution.

**Proof**

Let us fix any 0 < *a* < *η* < *b* < 1. From conditions (56)–(58) in the proof for Theorem 4 we can see that

(79) fx≥f0∧fa>0if  0<x≤a,fx≤fb<0if  b≤x<1.

Let us now define the stopping time *τ* as we did in Lemma 6. Recall that

(80) log⁡πt=log⁡π0+∫0tfπsds+∫0t1−πsυ¯σπsτ+ρ−πsρdBs,

and then, by using (79), we have that, for all *t* ≥ 0 and for any *π*_0_ ∈ (0, *a*),

(81) log⁡aElog⁡πt∧τ≥log⁡π0+f0∧faEt∧τ,

and then

(82) $$\begin{array}{c} \log\left(\;\frac{a}{\pi_{0}}\;\right)\geq \left(\;f\left(\;0\;\right)\wedge f\left(\;a\;\right)\;\right)E\left(\;t\wedge \tau \;\right). \end{array}$$

By letting *t* → *∞*, we have that for all *π*_0_ ∈ (0, *a*)

(83) $$\begin{array}{c} E\left(\;\tau \;\right)\leq \frac{\log\left(a/\pi_{0}\right)}{f\left(0\right)\wedge f\left(a\right)}. \end{array}$$

Similarly, for any *π*_0_ ∈ (*b*, 1), we have that

(84) log⁡bElog⁡πt∧τ≤log⁡π0−fbEt∧τ,∀t≥0,

and then

(85) $$\begin{array}{c} \log\left(\;\frac{b}{\pi_{0}}\;\right)\leq -\left|\;f\left(\;b\;\right)\;\right|E\left(\;t\wedge \tau \;\right). \end{array}$$

By letting *t* → *∞*, we have that

(86) $$\begin{array}{c} E\left(\;\tau \;\right)\leq \frac{\log\left(\pi_{0}/b\right)}{\left|f\left(b\right)\right|}\leq \frac{\log\left(1/b\right)}{\left|f\left(b\right)\right|}\;\forall \pi_{0}\in \left(b,1\right). \end{array}$$

Clearly, the conditions required for existence of a unique stationary distribution mentioned in Lemma 6 are satisfied by (83) and (86) and thus we have completed our proof and our SDE HIV model (14) has a unique stationary distribution.

## 7. Simulations

In this section we will support our analytical results using numerical simulations produced in *R*. Throughout this section, various simulations are produced using realistic parameter values but our main objective is to verify the analytic results. Before we begin, let us make the same assumptions as in [5]. Without loss of generality, let us take *p* = 0 and assume that all PWIDs visit shooting galleries at the same rate whether or not they are infected and thus *λ*_1_ = *λ*_2_. In addition, we take *ϕ*_1_ = *θ*_1_ = 0 as these probabilities are very small.

### 7.1. Simulations on Extinction

In this section, we will focus on looking at the numerical simulations produced which support the analytical results given in Theorems 2 and 3.

**Example 8 ($R_{0}^{S}<1,\;{\bar{\upsilon }}^{2}\leq \upsilon \rho /\sigma$). **

Let us choose realistic parameter values *μ* = 0.258/year = 7.06849 × 10^−4^/day [7], *λ*_1_ = *λ*_2_ = 0.143, *α* = (*P*_1_ + *P*_3_) = 0.01, *θ* = (*P*_1_ + *P*_2_) = 0.25, *γ* = 1 (based on [5]), and *ξ* = 0.6 [10]; then, from (1) and (4), we have that *σ* = 0.0572, *τ* = 0.143, *ρ* = 0.1001, and *υ* = 0.00143. Then by choosing $\bar{\upsilon }=0.046$, we have that

(87) $$\begin{array}{c} {\bar{\upsilon }}^{2}=0.002116<\frac{\upsilon \rho }{\sigma }=0.0025025, \end{array}$$

where *R*_0_^*S*^ = 0.66729 < 1 while *R*_0_^*D*^ = 1.156. Therefore, by Theorem 2, we would expect the solution *π*(*t*) to reach zero with probability one.

**Figure 1:**
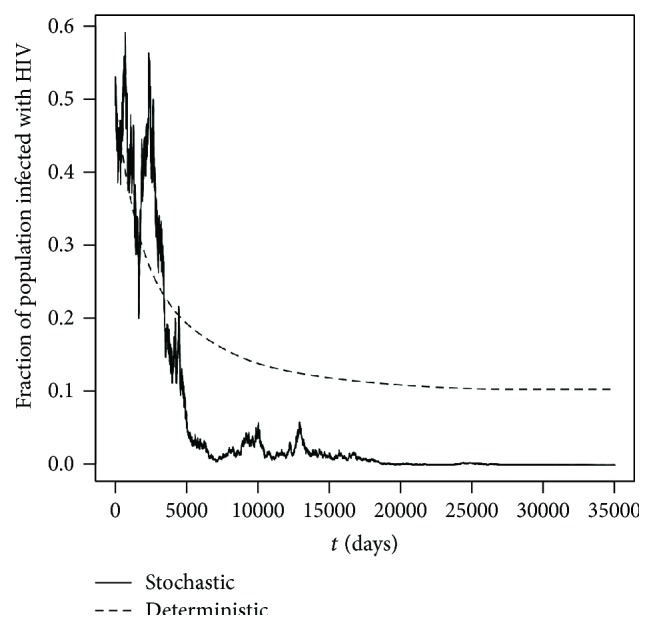
Computer simulations of the path *π*(*t*) for the SDE HIV model (14) and its corresponding deterministic HIV model (11) with step size Δ = 0.01 with parameter values given in Example 8 with initial value *π*(0) = 0.5.

The computer simulation produced in *R* using the Euler-Maruyama method ([21, 28]) with the above parameter values is given in Figure 1, which clearly illustrates that *π*(*t*) hits zero in finite time a.s. The numerical simulations were repeated numerous times with different initial value of *π*_0_ ∈ (0,1) and similar results were obtained each time.

**Example 9 ($R_{0}^{S}<1,\;{\bar{\upsilon }}^{2}>\upsilon \rho /\sigma \vee \upsilon^{2}/2\mu$). **

By using the same parameter values as in Example 8 but choosing $\bar{\upsilon }$ to be 0.07 and thus ${\bar{\upsilon }}^{2}=0.0049$, we have that

(88) $$\begin{array}{c} {\bar{\upsilon }}^{2}>\frac{\upsilon \rho }{\sigma }\vee \frac{\upsilon^{2}}{2\mu }, \end{array}$$

where *R*_0_^*S*^ = 0.02425249 < 1 while *R*_0_^*D*^ = 1.156035. As a result, by Theorem 3, we could conclude that, for any initial value *π*(0) = *π*_0_ ∈ (0,1), the solution *π*(*t*) obeys

(89) $$\begin{array}{c} \underset{t\to \infty }{lim}\sup\frac{1}{t}\log\left(\;\pi \left(\;t\;\right)\;\right)\leq -0.000498186<0\;a.s. \end{array}$$

**Figure 2:**
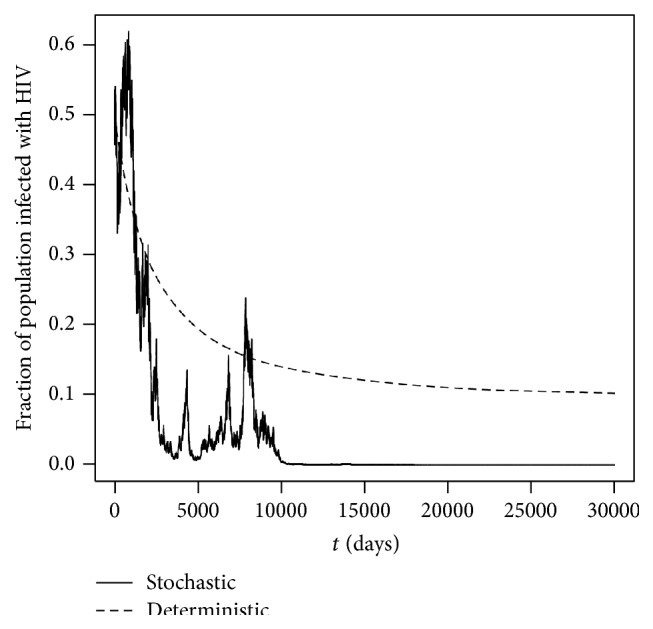
Computer simulations of the path *π*(*t*) for the SDE HIV model (14) and its corresponding deterministic HIV model (11) with step size Δ = 0.01 using parameter values given in Example 9 with initial value *π*(0) = 0.5.

Clearly Figure 2 supports this result by showing that the solution *π*(*t*) reaches zero at finite time. Again, the numerical simulations were repeated several times with different initial values and the same results were concluded.

### 7.2. Simulation on Persistence

We will now move on to the numerical simulations for results given in Theorem 4 and Proposition 5.

**Example 10 (*R*_0_^*S*^ > 1).**

Let us use the same parameter values as in Example 8 but changing *μ* to 0.125/year and thus 3.42466 × 10^−4^/day [4]. Let us define $\bar{\upsilon }=0.05$ and thus *R*_0_^*D*^ = 2.386057 and *R*_0_^*S*^ = 1.1942 > 1. Therefore by Theorem 4, for any given initial value *π*_0_ = *π*(0)∈(0,1), the solution *π*(*t*) for the SDE HIV model (14) should obey

(90) $$\begin{array}{c} \underset{t\to \infty }{lim}\inf\pi \left(\;t\;\right)\leq \eta =0.3206092\leq \underset{t\to \infty }{lim}\sup\pi \left(\;t\;\right)\;a.s. \end{array}$$

**Figure 3:**
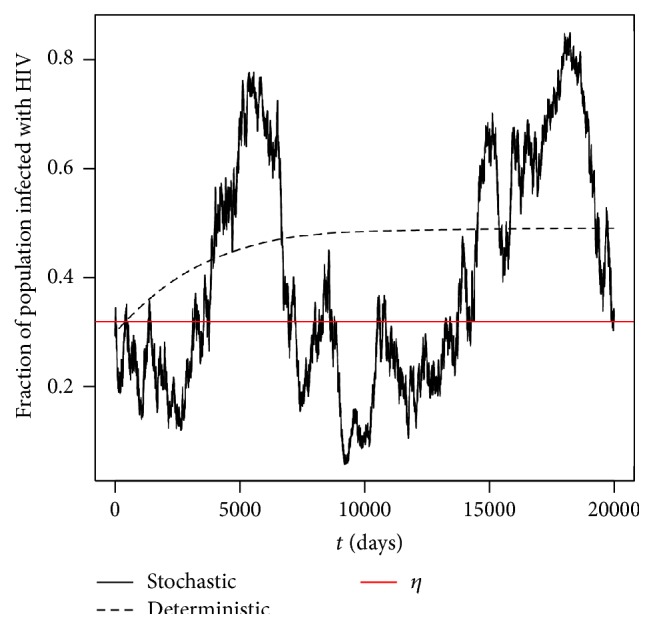
Computer simulations of the path *π*(*t*) for the SDE HIV model (14) and its corresponding deterministic HIV model (11) with step size Δ = 0.01 using parameter values given in Example 10 with initial value *π*(0) = 0.3.

Figure 3 clearly supports our analytical results given in Theorem 4 by showing the solution path of *π*(*t*) oscillates around the level *η* in finite time. Again the numerical simulations were repeated and the same conclusion can be drawn each time.

In order to further illustrate the effect of the noise intensity $\bar{\upsilon }$ has on the solution, in the next example we will keep all the parameter values the same as in Example 10 but reducing the noise intensity.

**Example 11 .**

By keeping the parameter values the same as in Example 10 and reducing $\bar{\upsilon }$ to 0.02, we have that *R*_0_^*D*^ = 2.38605, *R*_0_^*S*^ = 2.195363 > 1, and *η* = 0.4770654. By Theorem 4 and Proposition 5, we would expect the solution *π*(*t*) to persist and oscillate around the level *η*. Furthermore by Proposition 5, as $\bar{\upsilon }\to 0$, we would expect *η* to tend towards the deterministic equilibrium value for the corresponding deterministic model given by (11); namely, (*υσ* − *μρ*)/(*υσ* − *μρ* + *μτ*) = 0.4924476.

**Figure 4:**
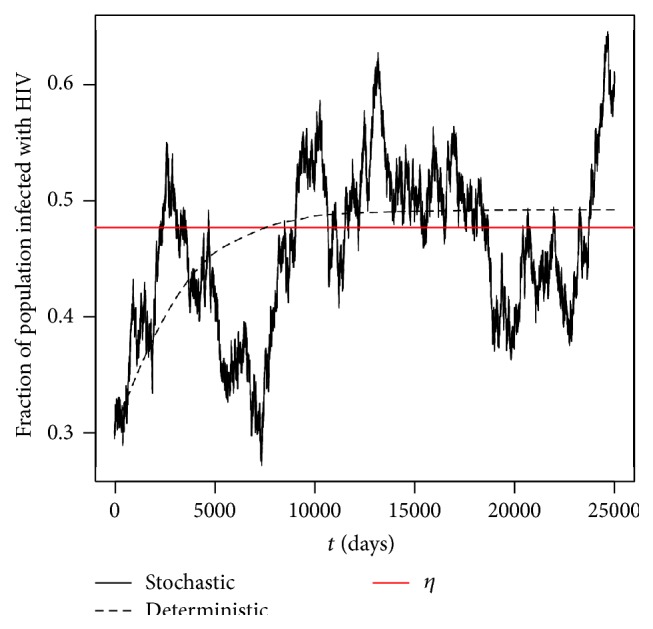
Computer simulations of the path *π*(*t*) for the SDE HIV model (14) and its corresponding deterministic HIV model (11) with step size Δ = 0.01 using parameter values given in Example 11 with initial value *π*(0) = 0.3.

From Figure 4, we can clearly see that the solution path *π*(*t*) does indeed oscillate about the level *η*. Moreover, by comparing Figures 3 and 4, we can also see that as we reduce the noise intensity from 0.05 to 0.02, the level *η* does indeed tend towards the deterministic equilibrium value as expected.

In the next example we will use histograms to see how the solution of the SDE HIV model oscillates around the level *η* as we vary the noise intensity $\bar{\upsilon }$.

**Example 12 .**

Let us use the same parameter values as in Example 10 and choose $\bar{\upsilon }$ to be 0.05, 0.04, 0.03, 0.005, and 0.001. We then let the simulations run for 1 million iterations but disregarding the first 800,000 iterations in order to allow *π*(*t*) to reach its recurrent level.

**Figure 5:**
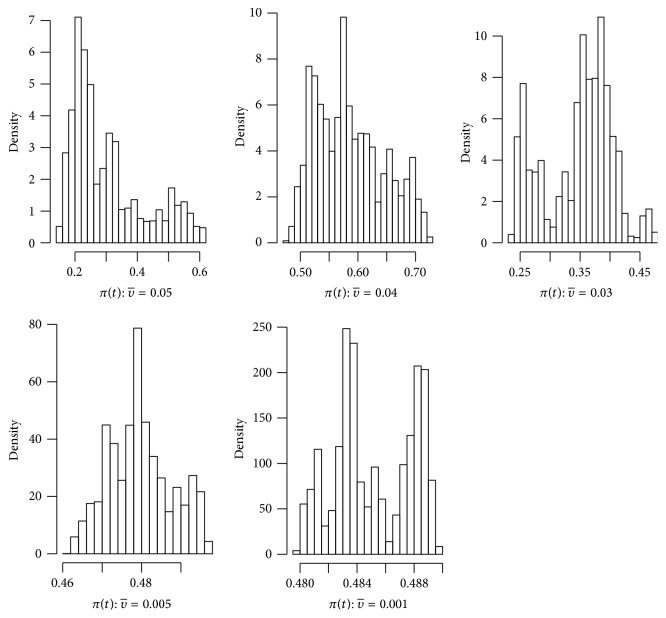
Histogram for the solution path *π*(*t*) for the SDE HIV model (14) with step size Δ = 0.01 using parameter values given in Example 12 with initial value *π*(0) = 0.3 and $\bar{\upsilon }=0.05,0.04,0.03,0.005$, and 0.001.

From Figure 5, we can see from the histograms that, for larger $\bar{\upsilon }$, the distribution of the solution is more skewed, while, for smaller $\bar{\upsilon }$, the distribution is more normally distributed about the level *η*. This is further confirmed by the sample skewness coefficients, namely, 1.000705, 0.4264459, −0.3415049, 0.2185976, and 0.003324568 corresponding to $\bar{\upsilon }=0.05$, 0.04, 0.03, 0.005, and 0.001, respectively.

**Figure 6:**
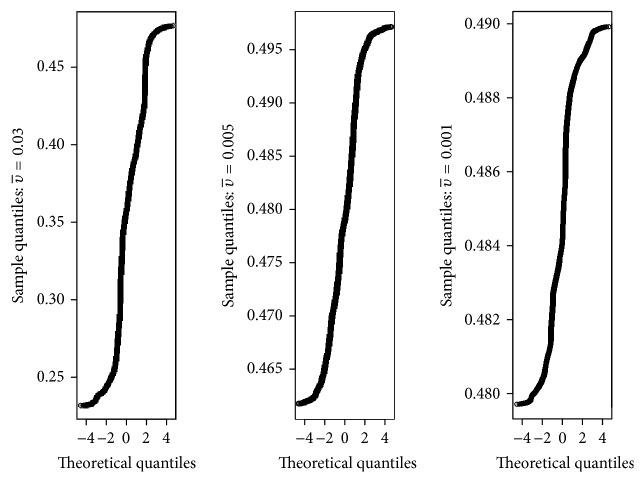
QQ plot for the solution path *π*(*t*) for the SDE HIV model (14) corresponding to the histograms shown in Figure 5 for $\bar{\upsilon }=0.03,0.005$, and 0.001.

Furthermore, we have used the quantile-quantile plot (QQ plot) to further illustrate that, for the smaller values of $\bar{\upsilon }$, these data are not far from being normally distributed. The result is shown in Figure 6.

## 8. Conclusion

In this paper we have introduced environmental stochasticity into the extended Kaplan model for the spread of HIV amongst PWIDs constructed by Greenhalgh and Hay [5]. Inspired by the work done on introducing stochasticity by parameter perturbation into the SIS epidemic model in [21], we explored the properties for the resulting stochastic HIV model by first proving that there exists a unique nonnegative solution *π*(*t*) for any given initial value *π*_0_ ∈ (0,1). Furthermore, we have constructed the basic reproduction number for the stochastic model, namely, *R*_0_^*S*^, and the conditions required for extinction and persistence for our solution *π*(*t*). In general, if *R*_0_^*S*^ < 1, the solution will almost surely go extinct as shown in Theorems 2 and 3. There is a gap in our results if *R*_0_^*S*^ and $\upsilon \rho /\sigma <{\bar{\upsilon }}^{2}<\upsilon^{2}/2\mu$ but here we conjecture that disease will always die out. This conjecture was supported by simulation. On the other hand, the solution will almost surely persist and oscillate around the level *η* if *R*_0_^*S*^ > 1 as shown in Theorem 4. Most importantly, we have shown that, by altering the noise intensity $\bar{\upsilon }$, it will affect the dynamical behaviour of our system.

By using the well-known Khasminskii theorem, we have shown that the SDE HIV model has a unique stationary distribution. Lastly, numerical simulations using realistic parameter values are constructed to support our analytical results.

Note that *R*_0_^*S*^ has a natural interpretation as follows: if we consider introducing a single newly infected individual into the disease-free equilibrium (DFE) and consider the number of secondary cases that he or she produces, then near the DFE equation (14) becomes

(91) $$\begin{array}{c} d\pi \left(\;t\;\right)=\pi \left(\;\frac{\upsilon \sigma }{\rho }-\mu \;\right)dt+\frac{\bar{\upsilon }\sigma }{\rho }\pi dB, \end{array}$$

with solution

(92) πt=π0exp⁡υσρ−μ−12υ¯2σ2ρ2t+υ¯σρBt.

Also lim_*t*→*∞*_|*B*(*t*)|/*t* = 0 a.s. Hence we expect that if

(93) $$\begin{array}{c} R_{0}^{S}=\left(\;\frac{\upsilon \sigma }{\mu \rho }\;\right)-\frac{{\bar{\upsilon }}^{2}\sigma^{2}}{2\rho^{2}\mu }<1, \end{array}$$

then the disease dies out whereas if *R*_0_^*S*^ > 1, the disease takes off. Thus this is a natural biological interpretation of the stochastic basic reproduction number *R*_0_^*S*^.

Deterministic models have in the past proved very useful in describing the spread of HIV amongst PWIDs but they have their faults. The real world is stochastic and in general stochastic models are more realistic than deterministic ones. Recall that

(94) $$\begin{array}{c} R_{0}^{S}=R_{0}^{D}-\frac{{\bar{\upsilon }}^{2}\sigma^{2}}{2\rho^{2}\mu }, \end{array}$$

where *R*_0_^*D*^ represents the basic reproduction number in the deterministic model. So in the deterministic model *R*_0_^*D*^ is the expected number of secondary cases caused by a single newly infected PWID entering a population consisting entirely of susceptible PWIDs and uninfected needles. The second term in (94) is an adjustment factor for the stochastic model.

In the deterministic model we have a straightforward scenario where if the basic reproduction number *R*_0_^*D*^ ≤ 1, then it is known that the disease will die out, whereas if *R*_0_^*D*^ > 1, then the disease will persist. The results in this paper show that, in the stochastic model, if *R*_0_^*S*^ < 1, then the disease dies out (almost surely), whereas if *R*_0_^*S*^ > 1, then the disease ultimately persists and oscillates about a nonzero level. These theoretical results are confirmed by numerical simulations. Moreover the argument above shows that if a single newly infected PWID enters the DFE, then we expect the disease to die out if *R*_0_^*S*^ < 1 and take off if *R*_0_^*S*^ > 1.

These findings provide new insights into the spread of HIV amongst PWIDs. Because the stochastic basic reproduction number *R*_0_^*S*^ is less than the deterministic one, it is possible for the noise to drive the disease to extinction, that is, if *R*_0_^*D*^ > 1, so that in the deterministic model the disease will persist; then if the stochastic noise is large enough, in the stochastic model the disease will die out. This has important implications for control strategies. Deterministic models have often been used to predict control strategies, for example, the fraction of PWIDs who must clean their needles after use, the effects of HIV testing, or the amount that PWIDs need to decrease their syringe sharing rates in order to reduce *R*_0_^*D*^ beneath one and eliminate disease. Examples of this applied to HIV amongst PWIDs include Greenhalgh and Lewis [32], Lewis [33] as well as Lewis and Greenhalgh [34]. Examples applied to hepatitis C virus (HCV) control include Corson [35] and Corson et al. [23].

The analytical and numerical results of this paper provide new insight into this. If there is significant stochastic noise in the system, then these estimates will be overestimated; that is, a smaller fraction of PWIDs cleaning their needles or a smaller reduction in PWID syringe sharing rates will still be sufficient for elimination of disease transmission.
